# Role of Single Procalcitonin Test on Admission as a Biomarker for Predicting the Severity of *Clostridium difficile* Infection

**DOI:** 10.3389/fmicb.2017.02532

**Published:** 2017-12-19

**Authors:** Zohar Hamo, Maya Azrad, Orna Nitzan, Asaf Sagie, Linda Tkhawkho, Dana Binyamin, Avi Peretz

**Affiliations:** ^1^Clinical Microbiology Laboratory, The Baruch Padeh Medical Center, Poriya, Tiberias, Israel; ^2^The Faculty of Medicine in the Galilee, Bar-Ilan University, Safed, Israel; ^3^Infectious Diseases Unit, The Baruch Padeh Medical Center, Poriya, Tiberias, Israel

**Keywords:** *Clostridium difficile*, procalcitonin, biomarker, score indices, antibiotic-associated diarrhea

## Abstract

**Objective:** To evaluate whether serum Procalcitonin (PCT) at the early stage of infection can serve as a potential biomarker for determining *Clostridium difficile* infection (CDI) severity.

**Methods:** Fifty-four patients diagnosed with CDI were enrolled in the study. Serum samples were obtained within a median time of 24–48 h of the lab result for presence of *C. difficile*. PCT levels were measured by chemiluminescence immunoassay. Demographic, clinical, and prognostic data concerning the patients were retrospectively collected from medical records. The illness severity score was determined according to “Score indices for *C. difficile* infection severity.”

**Results:** We found that serum PCT levels were significantly higher in patients with moderate disease, compared to patients with mild disease (*p* = 0.0032). Additionally, PCT was correlated with mortality (*p* = 0.0002), white blood cell count (*p* = 0.019), and community-acquired disease (*p* = 0.0345).

**Conclusion:** Early measurement of PCT may serve as a biomarker for early prediction of CDI severity, which is of great importance due to the high risk of complications and death.

## Introduction

*Clostridium difficile* is a gram positive, toxin-producing, anaerobic rod that is the predominant etiological agent of antibiotic-associated diarrhea and pseudomembranous colitis (Bartlett et al., 1978). Additionally, it is the primary cause of nosocomial infections in hospitalized patients (Barbut and Petit, 2001).

The pathogenesis of CDI depends on several virulence factors contributing to disease development and severity; one main factor is the production of two toxins, toxin A and toxin B, glycosyltransferases that target Rho proteins. These cytosolic proteins are involved in various signaling processes including actin cytoskeleton regulation, cell cycle progression, and gene transcription, as well as regulation of various enzymes (Jank et al., 2007). In addition, these toxins lead to increased intestinal permeability and fluid accumulation resulting in diarrhea, which is the hallmark of CDI (Nottrott et al., 2007).

During the last decade, the incidence, severity, and recurrence of CDI have considerably increased, particularly with the emergence of hypervirulent strains (Stabler et al., 2009; Khanna et al., 2012), and yet it is unclear why only some patients develop severe disease. An early and correct assessment of *C. difficile* disease severity is important for rapid and specific treatment administration.

Current severity scores have low specificity and/or sensitivity for CDI severity assessment; these scores are based on comorbidities, clinical manifestations, laboratory tests, and imaging studies. Therefore, it is necessary to find efficient biomarkers for early identification of bacterial infections, which can reduce unnecessary use of antibiotics and may also improve treatment quality.

Procalcitonin is a precursor peptide of calcitonin with a molecular weight of 13 kDa and comprised of 116 amino acids. It is encoded by the *CALC-1* gene, located on chromosome 11. It is secreted by cells in tissues with activated macrophages and by a variety of parenchymal cells in the liver, kidney, lung, and pituitary gland, but is mainly secreted from thyroid gland C cells. PCT secretion is induced uniquely by bacterial infections, particularly in cases of pneumonia and sepsis (Nobre et al., 2008; Schuetz et al., 2009). In these situations, high serum PCT levels can be measured. In contrast, viral infections result in down regulation of PCT synthesis, which is mediated by interferon-γ (Linscheid et al., 2003; Becker et al., 2004, 2008).

Most importantly, serum PCT levels in healthy people are below the detection threshold (10 pg/ml). PCT levels above 2 ng/ml point to an acute bacterial infection, while in severe bacterial infections and sepsis PCT levels can rise to 10–100 ng/ml (de Jong et al., 2016; Trásy et al., 2016). Thus, PCT measurement can be helpful for distinguishing between bacterial infections and other inflammatory conditions (Christ-Crain and Müller, 2007).

Studies have shown that, compared with the known biomarkers currently in use, such as C-reactive protein (CRP), PCT has a higher sensitivity and specificity for severe sepsis and septic shock diagnosis (Simon et al., 2004; Kasperska-Zajac et al., 2013). Importantly, one of the major advantages of PCT is its biochemical stability in blood samples; therefore it can be tested along with routine samples without the need of special storage. Additionally, its half-life is 25–30 h.

Procalcitonin’s role in CDI is unknown. Current treatment strategy is to stratify severity of disease by clinical parameters and administer antibiotics according to disease severity. However, it is possible that clinical severity scores are not good enough in order to discriminate between mild, moderate, and severe disease. Therefore, it is of great importance in finding a biomarker that will assess the severity of CDI at an early stage of the disease. Our objective was to evaluate PCT as a potential alternative biomarker for determining CDI severity.

## Materials and Methods

### Study Population

Fifty-four patients were enrolled in the study at the Poriya Baruch Padeh Medical Center between November 2015 and May 2017. We excluded patients with pneumonia, sepsis of causes other than CDI, and patients with bacteremia.

All CDI cases were confirmed by stool examination for toxigenic *C. difficile*, identifying three targets: toxin B, binary toxin, and tcdC deletion presence using the GeneXpert *C. difficile* PCR assay (Cepheid, Sunnyvale, CA, United States). Blood samples were collected within 24–48 h of the positive diagnostic result and serum was separated by centrifugation.

The study was approved by the Poriya Baruch Padeh Medical Center Helsinki Committee. A signed informed consent for participation was obtained from all patients.

### Procalcitonin Measurement

Serum PCT levels were measured using the LIAISON^®^ BRAHMS PCT^®^ II GEN kit (Diasorin, Saluggia, Italy). The test is based on polyclonal antibodies binding to PCT in chemiluminescence immunoassay (CLIA) technology; a primary antibody conjugated to magnetic particles binds to PCT in serum samples. Then a secondary antibody conjugated to a photomere is added. A light reaction occurs through acid–base reactions.

Given the broad range of PCT values in our patients, we assigned undetectable PCT levels a value of 0.01 ng/ml and performed log_10_ transformation to the PCT values prior to statistical analysis.

### Disease Severity Scoring and Demographic Data Collection

*Clostridium difficile* infection patients were divided into mild, with a severity score index (SSI) of 0–3 points, and Moderate CDI, with an SSI of 4–7 points, based on a severity assessment score developed by Velazquez-Gomez et al. (2008) “Score indices for *Clostridium difficile* infection severity.” One point was given for each of the following parameters: changes in consciousness, pain/abdominal bulges, leukocyte levels above 20,000 μl/cells or below 1,500 μl/cells, albumin below 2.5 mg/dl, ascites/colitis, tachycardia above 110 ppm, mean arterial pressure (MAP) < 65 mmHg, transfer to Intensive Care Unit (ICU).

The following demographic data was retrospectively collected from the medical records: age, gender, community versus nosocomial acquired CDI, and death during hospitalization; as well as clinical data concerning the patients and creatinine levels.

### *In Vitro* Antibiotic Susceptibility Testing (AST)

The stool samples were cultured on chromID^TM^ *C. difficile* (bioMérieux, Durham, NC, United States) growth medium and then incubated at 37°C in anaerobic conditions for 48 h. *C. difficile* colonies appear asymmetric and black-colored (Eckert et al., 2013). Isolated colonies were suspended for generation of 1 McF turbidity. The inoculum was sub-cultured on Brucella Blood Agar (Becton Dickinson, Heidelberg, Germany), supplemented with hemin and vitamin K, and an Etest strip for metronidazole and Vancomycin (bioMérieux, Durham, NC, United States) was added. The agar plates were incubated with GasPak sachet for the generation of anaerobic conditions at 37°C for 24 h. Minimum inhibitory concentration (MIC) determination was performed in accordance with the European Committee on Antimicrobial Susceptibility Testing (EUCAST) guidelines; *C. difficile* isolates were considered as resistant when MIC was above 2 mg/L to metronidazole or Vancomycin (Adler et al., 2015).

### Toxins Detection

The presence of toxins A and B was tested using the CerTest *C. difficile* GDH+ Toxin A+ Toxin B kit (Certest Biotec, S.L, Spain). The kit is based on an immune-chromatographic assay for the qualitative detection of *C. difficile* antigen, Glutamate Dehydrogenase (GDH), and toxins A and B from a stool sample; test strips A, B, and C are coated with mouse monoclonal antibodies against toxin A, toxin B, and GDH, respectively. The sample is mixed with a test solution, which contains mouse monoclonal antibodies anti-GDH/Toxin A/Toxin B, conjugated to red polystyrene latex. When GDH antigen/Toxin A/Toxin B is present in the sample, the antigen/toxin reacts with the specific antibodies in the test solution, and this complex is captured by the antibodies in the test strips, resulting in a visible red line.

### Statistical Analysis

Chi-square or Non-parametric Wilcoxon-Mann-Whitney Rank sum test for independent samples were applied for analyzing the differences in distribution of categorical or continuous parameters, respectively, between mild and moderate disease severity.

The Non-parametric Wilcoxon-Mann-Whitney Rank sum test or analysis of variance (ANOVA) was applied for analyzing differences in categorical parameters between CDI score and PCT.

Pearson correlation was applied to test the correlation between CDI score (0–7) or PCT with other quantitative parameters.

Multivariate analysis was applied using Logistic Regression model, which examined the parameters predicting disease severity with adjustment to other covariates.

All tests applied were two-tailed, and a *p*-value of 5% or less was considered statistically significant. The data was analyzed using the SAS^®^ version 9.3 (SAS Institute, Cary, NC, United States).

## Results

Among the 54 CDI subjects enrolled in the study, 38 cases had mild CDI and 16 had moderate CDI. **Table 1** summarizes the demographic characteristics of patients from both groups. There were no significant differences between the two groups. **Table 2** presents the clinical data of these patients. Mean white blood cell (WBC) count in the moderate CDI group was 20,438.75 cells/μl (±1700–70,000 cells/μl) which was higher than the mean WBC count in the mild CDI group of 13,851.58 cells/μl (±2490–31,840 cells/μl (*p* = 0.0448). Mean albumin level in the moderate CDI group was 2.24 g/dl, compared to 2.69 g/dl in the mild disease group (*p* = 0.0473).

**Table 1:** Summary of the demographic characteristics of CDI patients, divided to mild and moderate severity disease groups.

	Mild disease (*n* = 38)	Moderate disease (*n* = 16)	
	
Characteristic	Mean (%)	Mean (%)	*p*-Value
**Gender**			
Male	16 (76.2)	5 (23.8)	
Female	22 (66.7)	11 (33.3)	0.455
**Toxin production**			
Toxin A	5 (83.3)	1 (16.7)	
Toxin B	8 (57.1)	6 (42.9)	
A+B	25 (73.5)	9 (26.5)	0.4023
**Infection acquisition**			
Nosocomial	29 (78.4)	8 (21.6)	0.0573
Community	9 (52.9)	8 (47.1)	
**In hospital mortality**			
Alive	33 (84.6)	6 (15.3)	
Died	5 (33.3)	10 (66.6)	**0.0002**


*Bold values indicate statistical significance, *p*-value < 0.05.*

There was a trend for higher CRP levels in the moderate (84.62 mg/l) versus the mild (65.26 mg/l) CDI group (*p* = 0.067) (**Table 2**).

**Table 2:** Clinical and laboratory parameters of CDI patients divided to mild and moderate severity disease groups.

	Mild disease (*n* = 38)		Moderate disease (*n* = 16)		
Clinical parameter	Mean	Range	Mean	Range	*p*-Value
Age (year)	77.05	46–98	75.38	58–92	0.6781
WBC [cells/μl]	13851.58	2490–31840	20438.75	1700–70000	**0.0448**
CRP [mg/l]*	65.26	6.41–100	84.62	31.6–100	0.067
Albumin [g/dl]	2.69	1.5–4.3	2.24	1.08–3.3	**0.0473**
Creatinine [mg/dl]	1.85	0.3–7.8	1.88	0.6–5.42	0.9623
PCT [ng/ml]	0.75	0.03–8.68	10.3	0.15–100	**0.0032**


*^∗^CRP > 100 was considered 100. Bold values indicate statistical significance, *p*-value < 0.05.*

**FIGURE 1:**
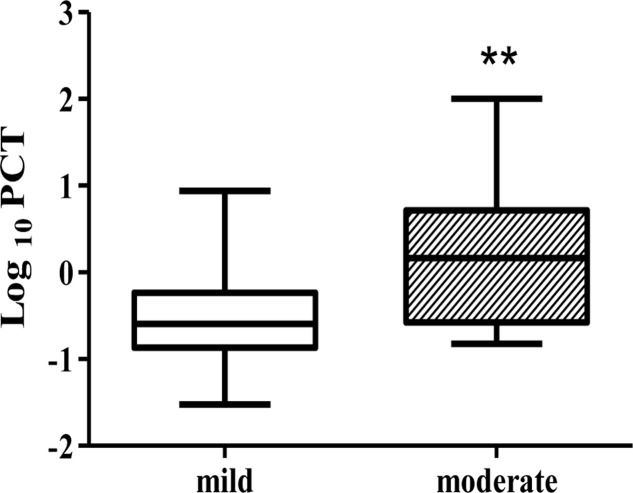
Procalcitonin (PCT) levels of patients with mild CDI and moderate CDI. The box plot shows the median (bold line), the first quartile (lower border of the box) and the third quartile (upper border of the box). ^∗∗^*p* < 0.01.

The mean PCT level was significantly higher in the moderate CDI group, 10.3 ng/ml (±0.15–100 ng/ml) than in the mild CDI group, 0.75 ng/ml (±0.03–8.68 ng/ml) (*p* = 0.0032) (**Figure 1**). We further investigated the correlation between PCT levels and the demographic and clinical parameters. A significantly positive correlation was found between PCT levels and community versus nosocomial acquired CDI (8.9 and 1.1 ng/ml, respectively, *p* = 0.0345) and with mortality at 30 days compared with patients who remained alive (11.2 and 0.6 ng/ml, respectively, *p* = 0.0002). In addition, there was a correlation between PCT and elevated peripheral WBC count (3.6 ng/ml, *p* = 0.019). No correlation was found between PCT serum levels and age, gender, toxins presence, CRP, albumin, creatinine levels, and resistance of strains to metronidazole and Vancomycin (**Table 3**).

**Table 3:** Correlation analysis between PCT and demographics and clinical parameters of CDI patients.

Parameter	*N*	PCT level [ng/ml] mean	*p*-Value
Age (year)	54	3.6	0.5512
**Gender**			
Male	21	2.9	
Female	33	4	0.4647
**Toxicity**			
Toxin A	6	0.3	
Toxin B	14	10.9	0.0768
A+B	34	1.2	
**Infection acquisition**			
Nosocomial	37	1.1	
Community	17	8.9	**0.0345**
**Metronidazole Susceptibility**			
Susceptible	44	4.3	
Resistant	10	0.6	0.6106
**Vancomycin susceptibility**			
Susceptible	52	3.7	
Resistant	2	1.5	0.3073
**In hospital mortality**			
Alive	39	0.6	
Died	15	11.2	**0.0002**
**Lab Parameters**			
WBC [cells/μl]	54	3.6	**0.0190**
CRP [mg/l]*	54	3.6	0.1651
Albumin [g/dl]	54	3.6	0.4434
Creatinine [mg/dl]	54	3.6	0.0842


*^∗^CRP > 100 was considered = 100. Bold values indicate statistical significance,*p*-value < 0.05.*

## Discussion

During the past decade, the clinical outcome of CDI has worsened, with increased numbers of cases and greater morbidity, and yet it is unclear why only some patients develop severe disease. An early and correct assessment of *C. difficile* disease severity is important for rapid and specific treatment administration.

There is great importance in finding a specific biomarker that could indicate the severity of disease. This objective assessment would allow for improved patient care. As an example, one study has found that elevated fecal calprotectin levels are associated with severity of CDI (Peretz et al., 2016).

In the current study we demonstrate that higher PCT concentrations are associated with a more severe CDI as assessed by the SSI. This means that high PCT levels are correlated with severity of disease and can play a role as a biomarker to predict the course of disease caused by *C. difficile*. This finding supports a previous report (Rao et al., 2013).

High PCT levels are also correlated with patient mortality. As mortality rate is higher in the moderate disease group, this result strengthens PCT compatibility as a marker for CDI severity.

These results might indicate that patients with higher PCT levels should be managed more aggressively compared with patients with lower PCT levels, which can be managed conservatively. PCT levels may also aid in choosing antibiotic treatment for CDI (Vancomycin versus metronidazole).

Additionally, we found that high PCT is significantly associated with elevated WBC count. WBC count was higher in patients with moderate disease. Possibly, moderate disease is characterized by more severe intestinal inflammation, resulting in increased WBC count. Thus PCT is not only correlated with the severity of CDI but also with one of the clinical manifestation of moderate disease, elevated WBC.

Recently, several studies have found an association between CRP and severity of bacterial infections. Our study did not find a significant correlation between CRP levels and CDI severity, although there was a trend for higher levels in patients with more severe disease. It might be that measurement of both markers, CRP and PCT, may have an increased sensitivity for detecting severe disease.

Patients with nosocomial disease had significantly lower PCT levels compared to patients with community-acquired (CA) disease. This finding was also observed in another study, which was conducted in France (Ogielska et al., 2015). We also found a trend for milder disease in cases of nosocomial infection than in CA-disease. It is possible that as CDI is a common hospital-acquired infection, these infections are usually recognized and treated early. In contrast, CA-CDI might be diagnosed at a more delayed stage of the disease in which PCT levels are much higher (Rao et al., 2013).

In our study we chose a certain scoring index. As other studies used different scoring methods (Peretz et al., 2016; Bauer et al., 2017), it is difficult to compare our results with those of other studies and thus there is a need to establish a uniform scoring system.

Our study has several limitations; first, we had a small sample size. Second, our study population didn’t include patients with severe CDI. A larger sample size, including patients with severe CDI, will help to strengthen our findings. In addition, we only measured PCT on admission, and did not follow kinetics of PCT levels, because we wanted to see if a single measurement on admission can predict disease severity and patient prognosis.

In summary, our findings indicate that PCT levels may predict disease severity of CDI. Measurement of this biomarker is easy and quick. It is important to recognize severe patients due to the high risk of complications and death.

In addition, future studies should compare PCT with other inflammatory markers such as cytokines and chemokines that may rise during CDI and may be correlated with disease severity.

A better and earlier assessment of illness severity will contribute to adjustment of medical treatment, including monitoring and follow-up.

## Author Contributions

ZH, AP, ON, and MA designed the experiments and wrote the paper. ZH, LT, AS, and DB performed the experiments and analyzed the data. All authors read and approved the final manuscript.

## Conflict of Interest Statement

The authors declare that the research was conducted in the absence of any commercial or financial relationships that could be construed as a potential conflict of interest.
